# The Non-Photosynthetic Algae *Helicosporidium* spp.: Emergence of a Novel Group of Insect Pathogens

**DOI:** 10.3390/insects4030375

**Published:** 2013-07-17

**Authors:** Aurélien Tartar

**Affiliations:** Division of Math, Science, and Technology, Nova Southeastern University, 3301 College Avenue, Fort Lauderdale, FL 33314, USA; E-Mail: aurelien@nova.edu; Tel.: +1-954-262-8148; Fax: +1-954-262-3931

**Keywords:** *Helicosporidium*, pathogenic algae, entomopathogens, *Prototheca*, Trebouxiophyte, biological control

## Abstract

Since the original description of *Helicosporidium parasiticum* in 1921, members of the genus *Helicosporidium* have been reported to infect a wide variety of invertebrates, but their characterization has remained dependent on occasional reports of infection. Recently, several new *Helicosporidium* isolates have been successfully maintained in axenic cultures. The ability to produce large quantity of biological material has led to very significant advances in the understanding of *Helicosporidium* biology and its interactions with insect hosts. In particular, the unique infectious process has been well documented; the highly characteristic cyst and its included filamentous cell have been shown to play a central role during host infection and have been the focus of detailed morphological and developmental studies. In addition, phylogenetic analyses inferred from a multitude of molecular sequences have demonstrated that *Helicosporidium* are highly specialized non-photosynthetic algae (Chlorophyta: Trebouxiophyceae), and represent the first described entomopathogenic algae. This review provides an overview of (i) the morphology of *Helicosporidium* cell types, (ii) the *Helicosporidium* life cycle, including the entire infectious sequence and its impact on insect hosts, (iii) the phylogenetic analyses that have prompted the taxonomic classification of *Helicosporidium* as green algae, and (iv) the documented host range for this novel group of entomopathogens.

## 1. Introduction

The genus *Helicosporidium* was first described by Keilin in 1921 [1]. The original specimen was isolated in England from the ceratopogonid larvae *Dasyhelea obscura* (Diptera), and named *Helicosporidium parasiticum*. In 1931, the genus and species names were validated and placed in a newly created order, Helicosporidia, in an attempt to classify these organisms [2]. The second report of *Helicosporidium parasiticum* in an insect host occurred in 1970, and interestingly, this pathogen was reported in a different insect host order (Lepidoptera) isolated from a different continent (Argentina) [3]. Albeit rare, *Helicosporidium* infections have since been reported from a variety of invertebrate hosts isolated from diverse geographic locations. In insect hosts, this pathogen has predominantly been found to infect larvae. As noted throughout the vast majority of reports, the unique morphological features of *Helicosporidium* facilitate identification, although it was recognized for a long time as a major obstacle to a precise taxonomic classification, especially during early descriptions [1,3].

## 2. The Cyst, Which Includes a Filamentous Cell, Is the Characteristic and Diagnostic Feature for the Genus *Helicosporidium*

**Figure 1:**
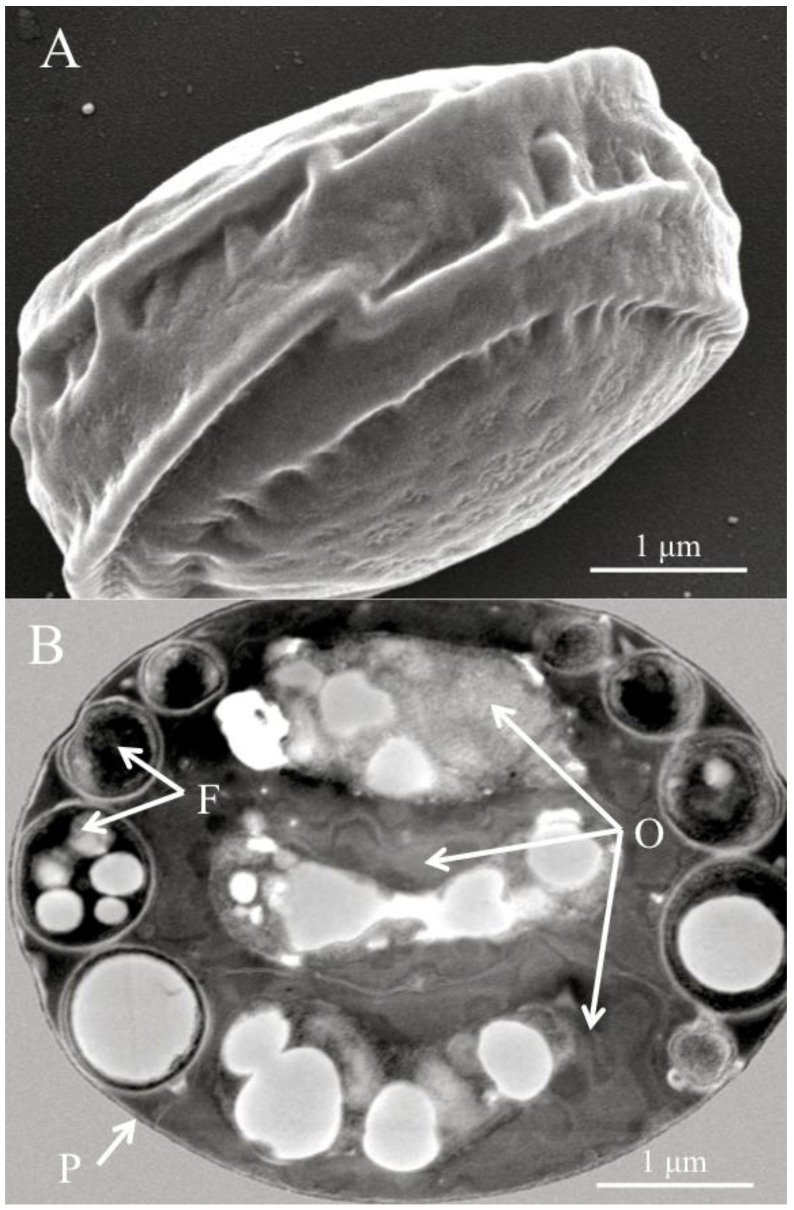
(**A**) Scanning electron micrograph of a diagnostic *Helicosporidium* cyst. (**B**) Transmission electron micrograph (cross section) detailing the core of three stacked ovoid cells (O) and the filamentous cell (F) contained within a pellicle (P).

Virtually all reports of *Helicosporidium* infection in invertebrate hosts rely on the observation of unique and characteristic four-cell structures [1,3,4,5,6,7,8,9,10,11,12,13]. These structures have been alternatively termed spores [1,3,4,5,6,7,9] or cysts [8,10,11,12,13]. Although the term spore was used during the original description of *Helicosporidium parasiticum* [1], and can still be found in occasional modern reports [9], most current studies, including this review, refer to the diagnostic feature of *Helicosporidium* as cyst. The *Helicosporidium* cyst is a barrel-shape structure that contains a core of three superposed ovoid cells surrounded by a single elongated, filamentous cell. These four cells are enclosed in a pellicle. The original *H. parasiticum* description featured elaborate drawings and microphotographs of the cyst and the filamentous cell [1]. Since then, electron microscopy photographs of the characteristic cyst have routinely been provided to support the identification of *Helicosporidium* in insect and other invertebrates. Recent reports of *Helicosporidium* sp. in various Coleopteran hosts, including the great European spruce bark beetle *Dendroctonus micans* (Coleoptera: Curculionidae), the predator beetle *Rhizophagus grandis* (Coleoptera: Rhizophaginae), and the weevil *Cyrtobagous salviniae* (Coleoptera: Curculionidae), all incorporated transmission electron micrographs (TEM) depicting cysts with the peripheral filamentous cell surrounding the three ovoid cells [10,11,12]. Cell measurements [7,8,10,12] indicated that the cysts are rather small, and range from 3 to 6 μm, although some of this variation might be due to differences in preparation methods [7]. Inside the cysts, the filamentous cell typically wraps around the core of ovoid cells three or four times, and can be distinguished on the narrow outer surface of the cysts (Figure 1). 

**Figure 2:**
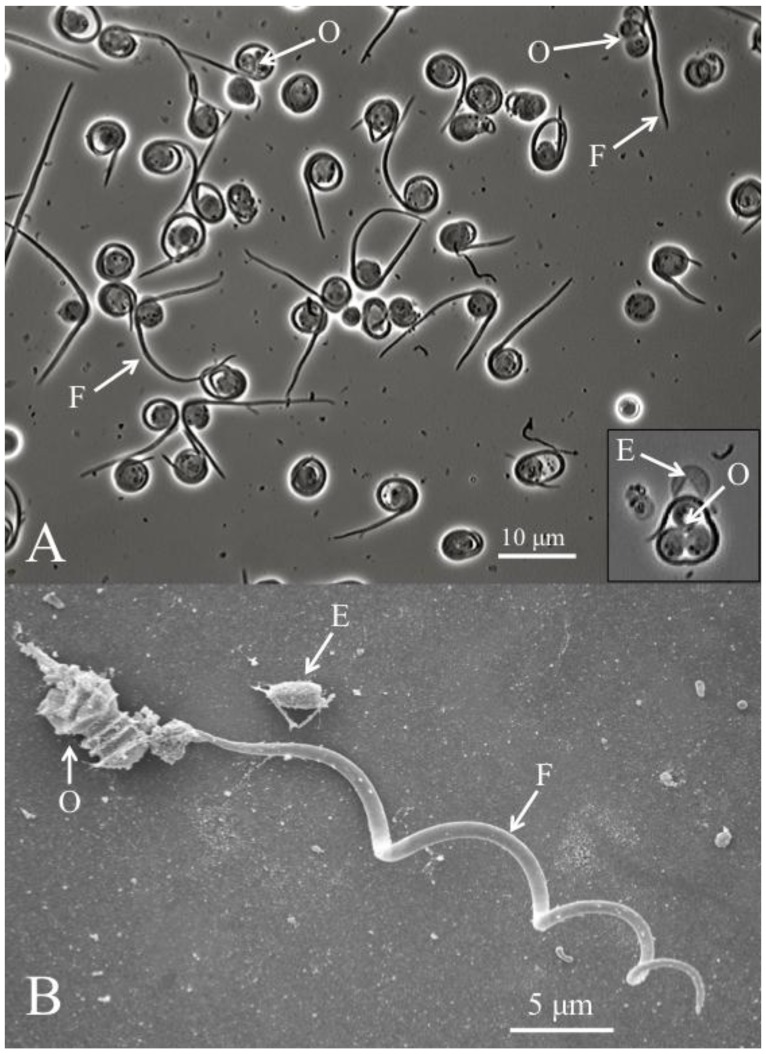
(**A**) *Helicosporidium* cyst dehiscence observed under light microscopy, showing groups of three ovoid cells (O), diagnostic filamentous cells (F) and empty pellicles (E). (**B**) Scanning electron micrograph detailing the filamentous cell and its barbs (pointing away from the core of ovoid cells).

In addition to TEM pictures, a recent study presented scanning electron microscopes pictures of cysts that were purified using Ludox gradient centrifugation, as well as light microscopy and SEM pictures of the filamentous cell being liberated from the cyst and separated from the remaining three ovoid cells [8]. This process is known as dehiscence (Figure 2). It has been observed both *in vitro*, by applying pressure to the microscope slide [8], and *in vivo*, in the gut lumen of susceptible hosts [14], and in the host hemolymph, after a series of desiccation events [1]. Significantly, dehisced cysts were used to single out the filamentous cells and highlight the presence of barbs [8] (Figure 2). Purified filamentous cells range in length from 37 to 62 μm [1,4,8,10,14]. It remains unclear if these ultrastructural differences can be related to the hosts from which these *Helicosporidium* were isolated.

The dividing stage of *Helicosporidium* corresponds to vegetative cells. These cells are characterized by the mitotic production of multi-cellular structures containing two, four or eight cells within a pellicle [14,15,16]. Similar to the cyst, the structure can rupture and release daughter cells from an empty pellicle. This mode of reproduction was termed autosporulation in reference to a similar process described for related taxa [16]. Meiosis has yet to be reported. Vegetative growth have been successfully obtained *in vitro* using cell-free artificial media, demonstrating that *Helicosporidium* can replicate without living host cells [8]. In heterologous, lepidopteran hosts, only a minority (15%–21%) of four-cell vegetative cells from a dipteran isolate developed into the typical cysts [16], and it is unknown if cyst morphogenesis is more efficient in natural hosts. Cyst differentiation has yet to be observed in *in vitro* cultures, suggesting that host–derived stimuli are required for this process [16]. Rather than developing into cysts, *Helicosporidium* cells allowed to undergo repetitive autosporulation cycles in artificial media were observed to form palmelloid colonies, which correspond to the agglutination of potentially abnormal vegetative cells [16]. Another notable difference between *in vitro* and *in vivo* vegetative development of *Helicosporidium* cells involves cell concentrations, as *in vitro* cultures obtained in artificial media failed to match the cell density observed in the host hemolymph [16].

Following the development of axenic cultures [8], three strains have been deposited the American Type Culture Collection (ATCC). These strains, labeled Sj-1, Dm-1, and Cs-1, refer to isolates, respectively collected from the black fly *Simulium jonesi*, the bark beetle *Dendroctonus micans*, and the weevil *Cyrtobagous salviniae* [8,10,12]. All three strains have been prudently named *Helicosporidium* sp., without a full species name, since it remains unclear if the genus is comprised of only one species, and if all *Helicosporidium* isolates are *Helicosporidium parasiticum* Keilin [1]. Comparative analyses between isolates are rare and have been complicated by difficulties in locating the original specimen. Hence, the vast majority of new *Helicosporidium* reports have been based on the limited description of the diagnostic cysts and filamentous cells, with little attempts to further the identification past the genus level, leading to reports of *Helicosporidium* sp. [6,7,8,9,10,11,12,13]. Polymorphic traits associated with cell morphology have been identified, but it is unclear if they are taxonomically significant [8,10,17]. Potentially, *in vitro* cultures of *Helicosporidium* may provide a basis to develop comparative analyses between isolates, and identify differences justifying the description of several *Helicosporidium* species. Comparative analyses of *in vitro* cultures of *Helicosporidium* Sj-1 and related taxa have already been performed [18]. In addition, axenic cultures will facilitate the characterization of the different cell phenotypes at the molecular level. In particular, the compositions of the *Helicosporidium* pellicle, or the vegetative cell wall, remain largely unknown, even if these structures play an important role in pathogenicity. Although molecular data has been generated in the form of expressed sequence tags (ESTs), they have been mainly used for phylogenetic analyses and not for molecular characterization of *Helicosporidium* cells [19]. To date, *in vitro* cultures have mostly proved crucial to obtain molecular sequences that precisely established the taxonomic affinities of *Helicosporidium* spp., and to investigate the transitions between cell types in order to fully understand the *Helicosporidium* life cycle.

## 3. The Cyst, and the Included Filamentous Cell, Initiates the *Helicosporidium* Infectious Cycle in Susceptible Insect Hosts

**Figure 3:**
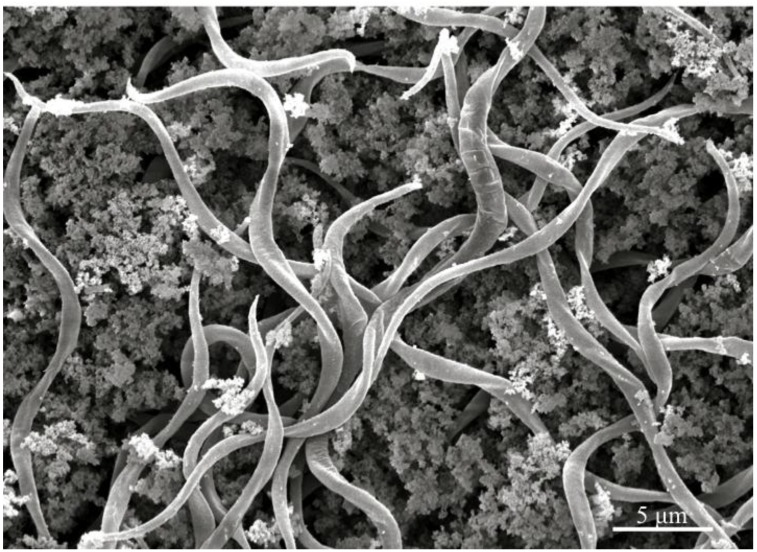
Scanning electron micrograph of the gut content of insect hosts challenged with *Helicosporidium* cysts, demonstrating that filamentous cells are released in the gut lumen.

The pathogenicity process of *Helicosporidium* spp. has been inferred principally from two long‑term studies that detailed the infection sequence in heterologous lepidopteran hosts maintained in laboratories [8,14,15,16,20]. A *Helicosporidium* originally isolated from larvae and adults of *Carpophilus mutilatus* (Coleoptera, Nitidulidae) was tracked in the navel orangeworm *Paramyelois transitella* [14,15]. Later, *Helicosporidium* Sj-1 was used to obtain infections in common laboratory insects such as the corn earworm *Helicoverpa zea*, the tobacco hornworm *Manduca sexta*, or the beet armyworm *Spodoptera exigua* [8,16,20]. Both studies established that cysts are the infective propagules and that infection occurs *per os*, when cysts are ingested by susceptible hosts. During insect bioassays, significant infection rates have routinely be obtained after *per os* challenge, even though a significant portion of intact cysts may be retrieved in the animal’s feces [20]. Gut dissections clearly demonstrated that cyst dehiscence was induced in the gut lumen [8,14,20]. The impact of insect gut on cysts was confirmed *in vitro*, as cysts incubated in midgut fluids were induced to rupture and release the filamentous cells [8]. Ovoid cells contained in the cysts were observed to lyse in the midgut [8,20]. *In vivo* cyst dehiscence is illustrated in Figure 3, complementing additional micrographs of insect gut lumen that demonstrated the infection process [8,20].

An initial investigation suggested that cysts might bind to the insect peritrophic membrane before dehiscence, and therefore be in a position where the released filamentous cells penetrate the peritrophic matrix immediately after dehiscence [8]. Although cyst dehiscence was initially described in the host hemocoel [1], it is now well established that filamentous cells are not found freely circulating in the hemolymph, but mediate the transition from midgut tissues to the host hemolymph, where *Helicosporidium* vegetative cells can be observed 24 h or 48 h post infection [8,14,16,20]. Detailed microscopic observations revealed that the released filamentous cells pass through the midgut epithelium, reach the hemocoel, and may create significant damage to the peritrophic membrane so that remaining cysts and cells gain access to the underlying tissues, and eventually, to the hemocoel [20]. Even the smallest described filamentous cells (37 μm) were estimated to be long enough to puncture the peritrophic membrane and the ectoperitrophic space, and gain access to the midgut cells [8]. The orientation of the filamentous cells barbs was found to be highly consistent, suggesting that the host‑pathogen interactions may be highly regulated at the cellular and molecular level. The barbs were first observed to be oriented towards the gut lumen as the filamentous cells initiated contact with the matrix, but then switched orientation and pointed towards the hemocoel as the filamentous cells gain ingress in the hemolymph [20].

The transition from invasive filamentous cells to replicating vegetative cells was elucidated recently under *in vitro* conditions [16]. In a manner similar to the more commonly observed autosporulation process, filamentous cells swell and undergo two division cycles within the original cell wall, releasing four bean-shaped daughter cells. These elongated cells divide again to produce spherical vegetative cells [16]. It is assumed that a similar differentiation takes place *in vivo*. Although uncommon, elongated cells were observed in both gut and hemolymph preparations of infected *P. transitella* [14]. Remnants of filamentous cells and bean-shaped cells were also observed being phagocytosed in infected *S. exigua* hemocytes [16]. The vegetative cells are responsible for hemolymph colonization. Since no filamentous cells are observed in the hemolymph, it is hypothesized that the transition occurs either in the host phagocytes for the filamentous cells that reach the hemolymph, or during the ingress from the gut tissue to the hemocoel, once the peritrophic matrix has been disrupted [20].

The vegetative cells are not recognized by the defense system of susceptible hosts, and replicate freely in the hemocoel, or in the hemocytes. They eventually fill the hemocoel, and have been showed to reach very high concentrations [16,20]. The *Helicosporidium* life cycle culminates as a portion of the vegetative cells differentiate into cysts [1,8,14,15,16,20,21]. This process has been observed both in the hemolymph and in hemocytes [20], and is impacted by both host age and pathogen dosage [21]. Although cyst production has yet to occur in artificial media, the vegetative cells obtained *in vitro* were shown to maintain their competence to differentiate into cysts when injected into host hemolymph, suggesting that a host-derived stimulus is required for cyst morphogenesis [16]. Direct injection of *Helicosporidium* cells in host hemolymph also resulted in 100% infection rates, which contrasted with the 50% rates reported for *per os* challenges [22]. This observation not only confirmed that cells are undetected by the host defense system, but also suggested that mechanisms of resistance against *Helicosporidium* infection are related to the ingress of the pathogen through the midgut [22].

High cell concentration and cyst morphogenesis in the hemolymph has variable impact on the insect host. There is no consistent or characteristic symptom associated with *Helicosporidium* infection in insects. Noticeable infection symptoms, such as change in hemolymph color and decreased host weight gain [23], or decreased host mobility [24], have occasionally been reported. Host death may be considered as the ultimate symptom, as suggested by the original *Helicosporidium* description [1]. However, several studies also indicated that the morphology [11,13] and the development (pupation) of infected insects [9,17,22,25] appeared unaffected. The lack of characteristic symptoms for *Helicosporidium* infection is consistent with the variable infectivity and mortality rates reported for this pathogen. Infection rates are dependent on both hosts and strains. A *Helicosporidium* sp. isolate tested on various mosquito species resulted in infection rates ranging from 0 to 93% [17]. In heterologous lepidopteran hosts, *Helicosporidium* Cs-1 produced an infection rate of 50% [22]. Bioassays demonstrated that infection rates are dose dependent [21,23]. Mortality rates have been equally variable, and can also be modulated by the host age and the concentration of cysts used to challenge potential hosts [21,26]. Significant mortality rates have been reported in several mosquito hosts, spurring substantial investigations on the potential of *Helicosporidium* as a biological control agent against mosquito larvae [9,17,24,25,26,27]. Overall, the variability in pathogenicity and virulence observed in different studies may also be correlated to the subsampled diversity within the genus *Helicosporidium*. The small number of infection bioassays that attempted to compare several *Helicosporidium* isolates all highlighted the fact that distinct isolates can be readily distinguished, based on differences in infectivity or virulence towards a single host [23,26]. These observations support the existence of several *Helicosporidium* species.

Lastly, although the *Helicosporidium* life cycle, from cyst ingestion to cyst morphogenesis in the hemolymph has been clearly established, no study has unambiguously described how *Helicosporidium* cysts exit the hemolymph of infected insects and are transmitted to subsequent hosts. The original *H. parasiticum* description indicated that infected larvae might become so impaired that the exoskeleton ruptures and the entire hemocoel content (including the infective cysts) is released into the environment [1]. This release has also been reported in mosquito larvae [26]. In pathosystems where the hosts appeared mostly unaffected and infection was trans-stadially maintained, vertical transmission of *Helicosporidium* was investigated. In mosquito hosts, infection of ovarian tissues was not observed [25], but vertical transmission was reported in noctuids [22]. Studies that described infections in both insects of a prey/predator system (the bark beetle *Dendroctonus micans* and its predator *Rhizophagus grandis*) suggested that *Helicosporidium* cysts may be directly transmitted by feeding when insects consume an infected prey, or potentially cannibalize an infected conspecies [10,11,13].

## 4. Phylogenetic and Phylogenomic Analyses Revealed that *Helicosporidium* spp. Are Non‑Photosynthetic Green Algae

The taxonomic classification for the genus *Helicosporidium* was historically a matter of debate, with these organisms alternatively proposed as Protozoa [1,2] or lower Fungi [3], based on morphological characteristics. However, the phylogenetic affinity of *Helicosporidium* has been unequivocally established by molecular-based analyses. The first sequenced gene fragments from *Helicosporidium* Sj-1 corresponded to rDNA, actin and β-tubulin. Phylogenetic reconstructions inferred from these genes revealed that members of the genus *Helicosporidium* are green algae (Chlorophyta) [28]. The SSU-rDNA gene fragment was also used to demonstrate that the genus *Helicosporidium* belongs to the chlorophyte class Trebouxiophyceae, and is closely related to the non‑photosynthetic algae of the genus *Prototheca* [28]. A set of 69 ribosomal protein sequences from the Sj-1 strain provided strong support, not only for the reclassification of *Helicosporidium* as green algae, but also for the specific relationship with *Prototheca *spp. [19]. Following this strongly supported, molecular-based taxonomic classification, similarities between *Helicosporidium* cells and trebouxiophyte green algae have also been highlighted at the cellular level, principally in regards to the vegetative cell multiplication and the autosporulation process [8,16].

The identification of *Helicosporidium* spp. as non-photosynthetic green algae prompted several studies aimed at characterizing chloroplast-like genetic material, since achlorophytic algae and plants have been shown to retain vestigial chloroplasts, or plastids. Amplification and analysis of plastid 16S rDNA gene fragments suggested that *Helicosporidium* cells had retained such an organelle [29]. Sequencing of a cluster of protein encoding genes demonstrated that the *Helicosporidium* plastid DNA was potentially transcribed [30], and a subset of expressed sequences tags annotated as homologs to nucleus-encoded, plastid-targeted proteins offered additional indirect evidence for a plastid and its included genome [31]. Although the organelle itself has yet to be observed in ultra-thin sections of *Helicosporidium* cells, the presence of a vestigial chloroplast has been thoroughly demonstrated by the sequencing of the entire plastid genome [32]. This genome lacks genes involved in photosynthesis and is so reduced that it is among the smallest known plastid genome. Overall, the plastid genome, and the phylogenetic inferred from its genes, have confirmed that *Helicosporidium* spp. are highly specialized non-photosynthetic trebouxiophyte green algae. Additional phylogenomic evidence was most recently provided by the analysis of the complete mitochondrial genome for *Helicosporidium* Sj-1 [33].

Trebouxiophyte algae include *Coccomyxa *spp., which are known pathogens of invertebrates such as mussels, scallops, geoducks, and starfishes [34,35,36]. However, *Coccomyxa* spp. are photosynthetic and have never been reported in insect hosts. The non-photosynthetic *Helicosporidium* spp. remain the only described achlorophytic entomopathogenic algae, and recent phylogenetic analyses indicated that the genera *Helicosporidium* and *Coccomyxa* are not closely related [37]. Virtually all phylogeny reconstructions have associated *Helicosporidium* with *Prototheca*, which harbors emerging pathogens of vertebrates, including isolates infecting humans, pets, and farm animals [38]. Both *Prototheca* and *Helicosporidium* are non photosynthetic, and since most phylogenetic analyses have depicted *Helicosporidium* and *Prototheca* as members of a monophyletic clade, it is hypothesized that the loss of photosynthesis represents a synapomorphic character for these algae. Only selected species of *Prototheca* are known to be pathogenic, and although there is similarities in cell division, pathogenic isolates of *P. wickerhamii* or *P. zopfii* have never been associated with a cyst stage, or filamentous cells, similar to *Helicosporidium*. To date, these unique cellular structures are thought to be specific to the genus *Helicosporidium* and, potentially, to its infectious process.

In addition to the studies aimed at precisely positioning *Helicosporidium* within a taxonomic framework, independent phylogenetic analyses focused on the genus *Prototheca* have incorporated the sequences generated from *Helicosporidium* spp. and confirmed the close relationship between the two genera [39,40]. Both *Prototheca* and *Helicosporidium* have appeared in a monophyletic clade that included the photosynthetic species *Auxenochlorella protothecoides*, and that has been referred to as the *Auxenochlorella*-*Helicosporidium*-*Prototheca* (AHP) clade [39]. In taxa-rich phylogenetic reconstructions focused on these genera, the species-level relationships have consistently been obscured by very poor resolution, and therefore the precise taxonomic position of *Helicosporidium* within the Trebouxiophyceae remains unclear. Lineages within the class Trebouxiophyceae are currently poorly resolved [41], and in the case of the AHP clade, it has been further complicated by the proposals for two novel *Prototheca* species, which have yet to appear in large-scale taxonomic classifications [42,43]. A general consensus might be emerging, as the inclusion of *Helicosporidium* has consistently led to phylogenetic trees that depicted *Prototheca* as a paraphyletic genus. Recent studies indicated that *Helicosporidium* is a sister taxon to a strongly supported clade that includes *P. zopfii*, *P. ulmea*, *P. moriformis*, *P. stagnora*, and the newly proposed *P. blaschkeae* [37,40]. Other species, such as *P. wickerhamii* and *P. cutis*, were not identified as members of this *Prototheca sensu stricto* clade, and therefore several authors have suggested that they should be re-assigned to alternative genera [39,40]. Although they have yet to include comparative analyses with *Helicosporidium*, alternative cladistic approaches, based on nutritional requirements [40], or biochemical profiles [38,39], have confirmed the strong heterogeneity of the genus *Prototheca*. To date, the closest relative to *Helicosporidium* spp. remain unidentified. A refined and potentially revised *Prototheca* taxonomy may be necessary to precisely establish species-level relationships between non‑photosynthetic trebouxiophyte algae, and determine if the acquisition of pathogenicity occurred once or multiple times in these algae.

Attempts to obtain strongly supported phylogenetic trees featuring both *Helicosporidium* and *Prototheca* have led to the generation of sequence data from multiple *Helicosporidium* isolates, and revealed an unsuspected genetic diversity. Sequences from plastid 16S rDNA genes [29], nuclear 18S rDNA and β-tubulin genes [36] contained polymorphic loci, suggesting that distinct *Helicosporidium* isolates might be differentiated at a molecular level. In phylogenetic trees, all *Helicosporidium* isolates were grouped in a strongly monophyletic clade, but analyses indicated that isolates collected from coleopteran hosts (strain Dm-1 and Cs-1) were more closely related to each other than they were to the dipteran isolate (strain Sj-1) [36]. Combined with the previously noted differences in relation to morphological structures, and infectivity and virulence, these polymorphic characters may serve as a basis to develop comprehensive studies aimed at distinguishing more than one *Helicosporidium* species. A refined understanding of the *Helicosporidium* biodiversity may help establishing whether the currently observed host range for these pathogens is reflective of one or more species.

## 5. The *Helicosporidium* Host Range Includes a Wide Variety of Insects and Other Invertebrates

Insect infections with *Helicosporidium* have been reported in three orders: Lepidoptera, Coleoptera and Diptera (Table 1). Overall, 23 species (11 families and 16 genera) of insects are known natural hosts for these pathogenic algae. Coleopteran and dipteran infections are most common. Only two instances of lepidopteran infections have been reported. A hepialid larva was first identified as a *Helicosporidium* host [3]. Later, *Helicosporidium parasiticum* was catalogued as a pathogen of the light brown apple moth (*Epiphyas postvittana) *in New Zealand [44]. In laboratory settings, lepidopteran larvae have been used extensively as heterologous hosts, clearly demonstrating that these insects are susceptible to infections [8,14,15,16,17,20,21,22,23,24].

**Table 1:** Reported insect host record for *Helicosporidium* spp.

Original insect hosts	Location	Heterologous insect hosts ^1^	Reference
**Diptera**			
Ceratopogonidae			
* Dasyhelea obscura*	England	nr	[1]
Rhiphidae			
* Mycetobia pallipes*	England	nr	[1]
Culicidae			
* Culex territans*	USA	nr	[45]
* Culex nigripalpus*	USA	Diptera (15) Coleoptera (3) Lepidoptera (2)	[26]
* Culex quinquefasciatus*	Thailand	nr	[24]
* Culex pipiens*	Egypt	Diptera (4) Lepidoptera (1)	[9]
* Aedes aegypti*	Thailand	Diptera (3) Lepidoptera (1)	[24]
Sciaridae			
* Ctenosciara hyalipennis*	Germany	nr	[46]
Simuliidae			
* Simulium jonesi*	USA	Diptera (4) Coleoptera (1) Lepidoptera (4)	[8]
**Coleoptera**			
Nitidulidae			
* Carpophilus dimidiatus*	USA	nr	[47]
* Carpophilus freeman*	USA	nr	[47]
* Carpophilus hemipterus*	USA	nr	[47]
* Carpophilus mutilatus*	USA, Mexico	Diptera (1) Coleoptera (9) Lepidoptera (5)	[47,48]
* Carpophilus pilosellus*	USA	nr	[47]
* Conotelus stenoides*	USA	nr	[47]
* Stelidota geminata*	USA	nr	[47]
* Urophorus humeralis*	Mexico	nr	[47]
Scarabaeidae			
* Orycetes monoceros*	Tanzania	nr	[49]
Curculionidae			
* Cyrtobagous salviniae*	South Africa	Diptera (3) Coleoptera (1) Lepidoptera (2)	[12]
* Dendroctonus micans*	Turkey	nr	[10]
Rhizophaginae			
* Rhizophagus grandis*	Turkey	nr	[11,13]
**Lepidoptera**			
Hepialidae			
* Hepialis pallens*	Argentina	nr	[3]
Tortricidae			
* Epiphyas postvittana*	New Zealand	nr	[44]

^1^ “nr” denotes that no heterologous hosts have been reported. When available, the numbers of heterologous host species is indicated in parenthesis.

Similar to the two infections reported in Lepidoptera, most coleopteran and dipteran accounts have corresponded to episodic observations (Table 1). However, notable exceptions exist. In Diptera, *Helicosporidium* infections have been recurrently reported in mosquitoes, especially in the genus *Culex*, which has been found as a host on four different occasions on various continents (North America, Asia, Africa), suggesting that *Helicosporidium* may be ubiquitous and chronic in mosquito populations [9,24,26,45]. Bioassays demonstrated that mosquito *Helicosporidium* isolates were infectious to additional mosquito species [9,26]. A large survey of nitidulid beetles pathogens suggested that *Helicosporidium* might be widespread in Coleoptera, as infections were reported in over 50% of the examined species [47]. A tentative identification in the rhinoceros beetle [49], combined with the recent detection of *Helicosporidium* in two members of the Curculionidae family [10,12] supports the possibility that coleopterans serve as major hosts for these pathogens. The first study aimed at investigating the abundance and importance of *Helicosporidium* on natural insect populations was recently performed on the coleopteran host *Dendroctonus micans*, and results indicated that the pathogen is widely distributed and can be detected over a period of three years in different geographic locales, with estimated infection rates reaching 71% [50]. A similar study focused on the occurrence of *Helicosporidium* in a second coleopteran host, the predator beetle *Rhizophagus grandis* (a predator of *D. micans*), although it was limited to laboratory-reared insect [51]. These two studies suggest that *Helicosporidium* investigations may move from isolated and occasional reports of infection in a few insects to a broader understanding of the abundance and impact of these organisms in insect populations.

In addition to the observation that *Helicosporidium* can be detected in Lepidoptera, Coleoptera and Diptera, one of the most striking and recurrent characteristics of these insect pathogens is arguably their ability to be horizontally transferred irrespectively of the host order from which they have been isolated. Isolates detected in dipteran hosts can readily infect coleopterans or lepidopterans [8,9,16,17,20,21,22,23]. Isolates collected in Coleoptera have showed similar broad host range, and were used to infect Lepidoptera or Diptera taxa [12,14,15,22,23,48]. The most comprehensive study of *Helicosporidium* host range involved an isolate collected from the nitidulid beetle *Carpophilus mutilatus* that was transmitted to nine other coleopteran species, five lepidopteran species and one dipteran species (Table 1) [48]. This isolate also failed to infect orthopteran and hymenopteran insects [48], providing the basis for, and supporting, the current understanding that the *Helicosporidium* host range is restricted to three insect orders: Coleoptera, Lepidoptera and Diptera, as indicated in Table 1. This study was also instrumental is demonstrating that the *Helicosporidium* host range includes non-insect invertebrates, as the coleopteran isolate was shown to be infectious to three species of mites. Mites and collembolans have been reported as potential natural hosts for *Helicosporidium*, as early as during the first description of *Helicosporidium parasiticum* [1,5,6,17,46]. In addition, *Helicosporidium* spp. were detected in trematodes [7] and cladocerans [4]. Overall, pathosystems involving non-insect hosts remain largely uncharacterized.

Finally, in agreement with the observation that the cuticle of heavily infected insect hosts may rupture and release *Helicosporidium* cysts and vegetative cells in the environment [1,52], several reports have indicated that *Helicosporidium* can be isolated [17,25,53], or at least detected [37], from lentic water samples such as ponds or ditches. Pathogenicity of these isolates was evaluated in the corn earworm *Heliothis zea*, or mosquitoes [17,25,53], suggesting that pathogenic isolates can be uncovered independently of host infections. When tested on mosquitoes, a *Helicosporidium* isolate collected from field water samples was compared to the Thailand mosquito isolate (from *Aedes aegyptii*), and was shown to be not as effective or as lethal [17]. This result prompted the proposal for distinct species of *Helicosporidium*, and was reminiscent of other comparative studies that indicated that pathogenicity to *Culex pipiens quinquefasciatus* was variable [26]. Although a *Helicosporidium* isolate from a coleopteran host was reportedly infectious to *Cx. p. quinquefasciatus* [48], this mosquito species appeared non-susceptible to an isolate collected from *Cx. nigripalpus* [26]. Although rare, the noted differences in host susceptibility and *Helicosporidium* virulence suggest that the list of natural hosts presented in Table 1 may be reflective of the combined range of several isolates that have yet to be formally distinguished and taxonomically separated.

As noted previously [37], the ability to consistently detect *Helicosporidium* in selected environments [17,25,37,53] or insect populations [50,51] will likely further the current understanding on these organisms by not only providing robust ecological data to estimate their impact on host populations but also expanding the current knowledge of *Helicosporidium* biodiversity and the identification of polymorphic characters that may be informative for the distinction of several *Helicosporidium* species.

## 6. Conclusion

The protists *Helicosporidium* spp. represent the first described entomopathogenic non‑photosynthetic algae. They are characterized by unique and diagnostic cell phenotypes that primarily include the infective cyst, a four-cell structure that is ingested by susceptible hosts and releases a filamentous cell that mediates host ingress and infection. Susceptible hosts include insects belonging to the Diptera, Coleoptera and Lepidoptera orders, as well as non-insect invertebrates such as mites and collembolans. Techniques associated with maintaining *Helicosporidium* spp. in axenic cultures *in vitro* and evaluating pathogenicity in insect bioassays have been well established. Despite the recent advances in understanding *Helicosporidium* biology, several challenges remain. One of these challenges involves characterizing the molecular basis of *Helicosporidium*-host interactions, and identifying the host factors potentially responsible for both cyst dehiscence in the gut, and cyst morphogenesis in the host hemocoel. Comprehensive investigations on the molecular composition of *Helicosporidium* cell phenotypes may shed light on the role of the filamentous cell barbs during ingress, or the ability of the vegetative cell wall to evade host detection systems. The filamentous cell is the most diagnostic feature of *Helicosporidium*, but also represents an extremely unique structure in the green algae phylum. Genomic analyses may be aimed at identifying the genes associated with the development of these cells and determining if these genes share a green algal origin. A second challenge includes developing a more global estimation of the occurrence and abundance of *Helicosporidium* in insect populations. Increasing the number of isolates available for comparative analyses will undoubtedly lead to an improved understanding of *Helicosporidium* diversity, refined trebouxiophyte phylogenies, and as noted throughput this review, may lead to the distinction of multiple *Helicosporidium* species.
